# Supplementary material for: The Ginsenoside Compound K Suppresses Stem-Cell-like Properties and Colorectal Cancer Metastasis by Targeting Hypoxia-Driven Nur77-Akt Feed-Forward Signaling
Source: Cancers (Basel). 2022 Dec 20;15(1):24. doi: 10.3390/cancers15010024 (PMC9817892; doi:10.3390/cancers15010024)
Supplement: Supplementary file 1 [file cancers-15-00024-s001.zip › cancers-2028167-supplementary.pdf]

Supplementary Materials: The Ginsenoside Compound K Suppresses Stem-Cell-like Properties and Colorectal Cancer Metastasis by Targeting Hypoxia-Driven Nur77-Akt Feed-Forward Signaling

Minda Zhang [1,†], Zeyu Shi [2,†], Shuaishuai Zhang [1], Xudan Li [1], Sally Kit Yan To [2], Yijia Peng [1], Jie Liu [1], Siming Chen [1], Hongyu Hu [3], Alice Sze Tsai Wong [2,*] and Jin-Zhang Zeng [1,*]

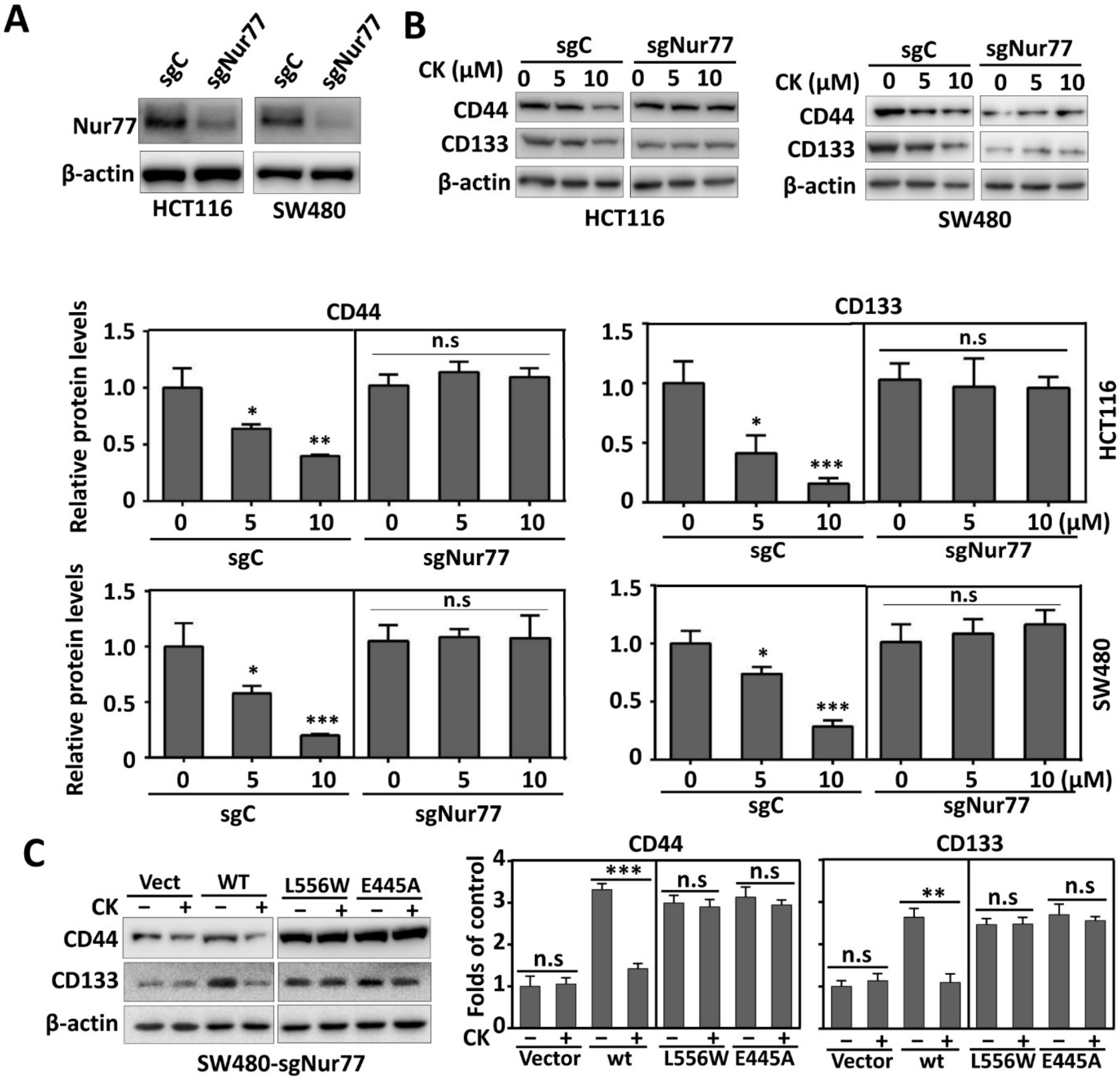

Figure S1. The supplementary figures for Figure 2. (**A**) Nur77 was stably knocked out in HCT116 and SW480 cells through CRISPR-Cas9. The efficiency of Nur77 knockout was tested by Western blotting. (**B**) Nur77/WT and Nur77/KO cells were treated with vehicle, 5 μM or10 μM of CK under hypoxic condition for 24 h. The protein levels of CD44 and CD133 were examined by Western blotting. (**C**) Nur77/KO cells were transfected with indicated Nur77 mutants for 24 h and treated with or without 10 μM CK for 24 h under hypoxic condition. The protein levels of CD44 and CD133 were examined by Western blotting. *$p < 0.05$; **$p < 0.01$; ***$p < 0.001$ vs respective control. Molecular weights for proteins were indicated in the full un-cropped annotated Western blot images (Figure S10).

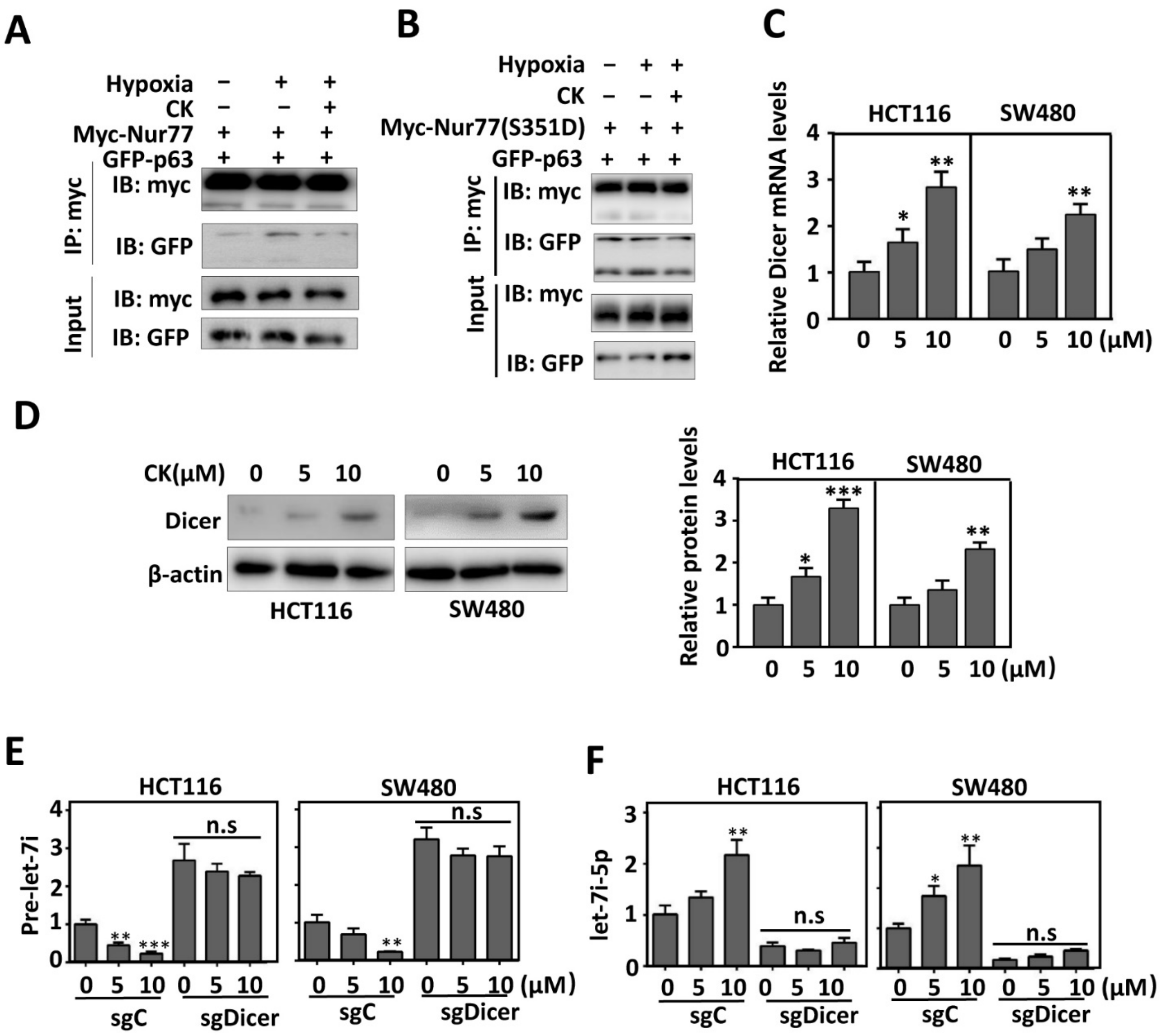

Figure S2. The supplementary figures for Figure 5 and 6. (**A**) HEK-293T cells were co-transfected with Myc-Nur77 and GFP-p63 for 24 h and treated with or without 10 μM CK under hypoxic condition for 4 h. Co-IP was performed with anti-Myc antibodies and blotted with anti-Myc and anti-GFP antibodies. (**B**) HEK-293T cells transfected with GFP-p63 and Myc-Nur77-S351D were treated with or without 10 μM CK for 4 h under hypoxia. Co-IP was performed with anti-Myc antibodies and blotted with anti-Myc and anti-GFP antibodies. (**C**) qPCR analysis of Dicer transcripts in CRC cells treated with vehicle or CK (5 μM or 10 μM) under hypoxic condition for 24 h. (**D**) The protein levels of Dicer were determined by Western blotting in CRC cells similarly treated as described in C. β-actin was served as an internal control. (**E–F**) Dicer/WT and Dicer/KO cells were treated with vehicle, 5 μM or 10 μM CK for 24 h under hypoxic condition. Levels of pre-let-7i and let-7i-5p were examined by qRT-PCR. *$p < 0.05$; **$p < 0.01$; ***$p < 0.001$ vs. respective control. Molecular weights for proteins were indicated in the full un-cropped annotated Western blot images (Figure S11).

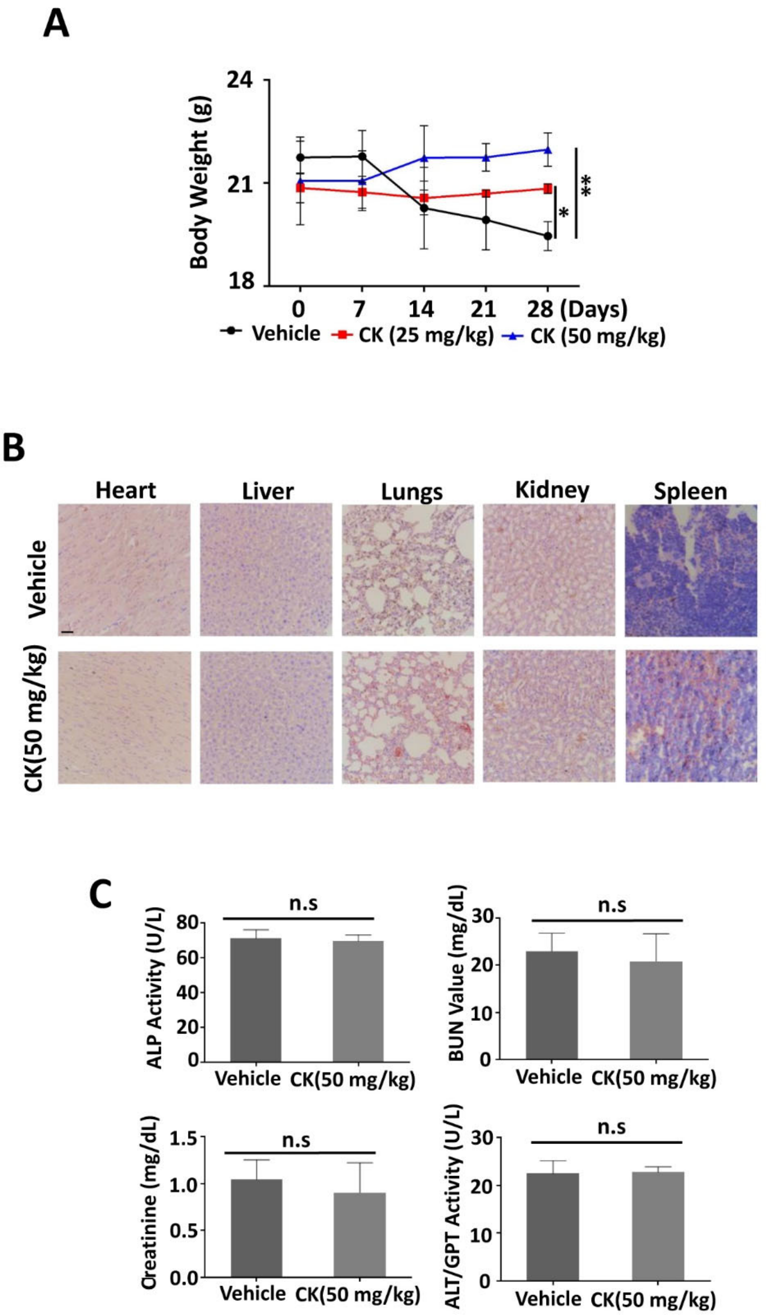

Figure S3. The supplementary figures for Figure 7. (**A**) The body weight of mice in each group shown. (**B**) Representative H&E staining images of heart, lungs, liver, spleen and kidney tissues from mice treated with 50 mg/kg CK. Scale bars: 100 μm in 10×. (**C**) Peripheral blood was collected from heart at sacrifice and alkaline phosphatase (ALP), alanine transaminase (ALT), creatine kinase, blood urea nitrogen (BUN) and creatine were measured and compared to vehicle injected mice. Data are shown as mean ± SD. *$p < 0.05$; **$p < 0.01$ vs. respective control.

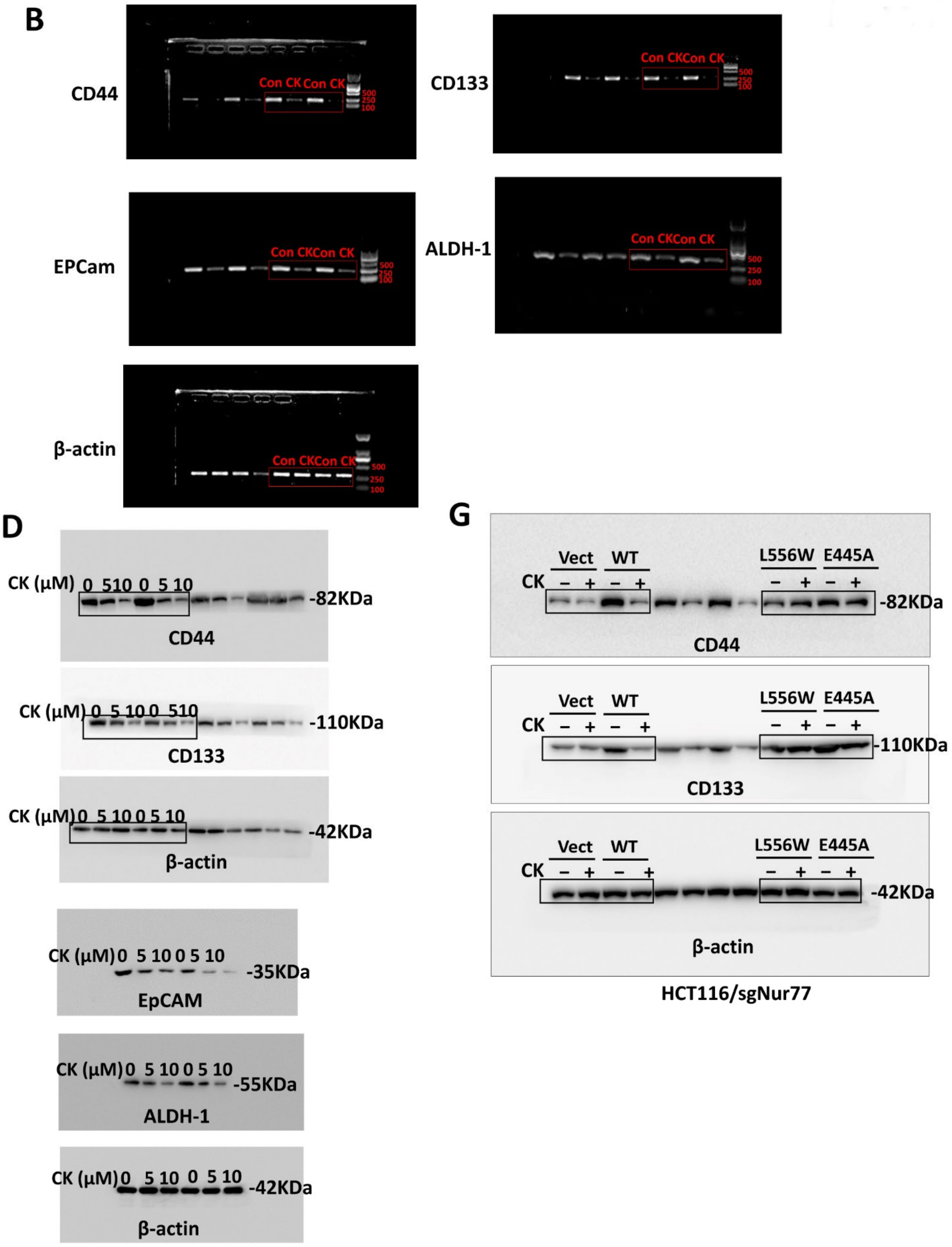

Figure S4. Uncropped Western Blot image for Figure 2.

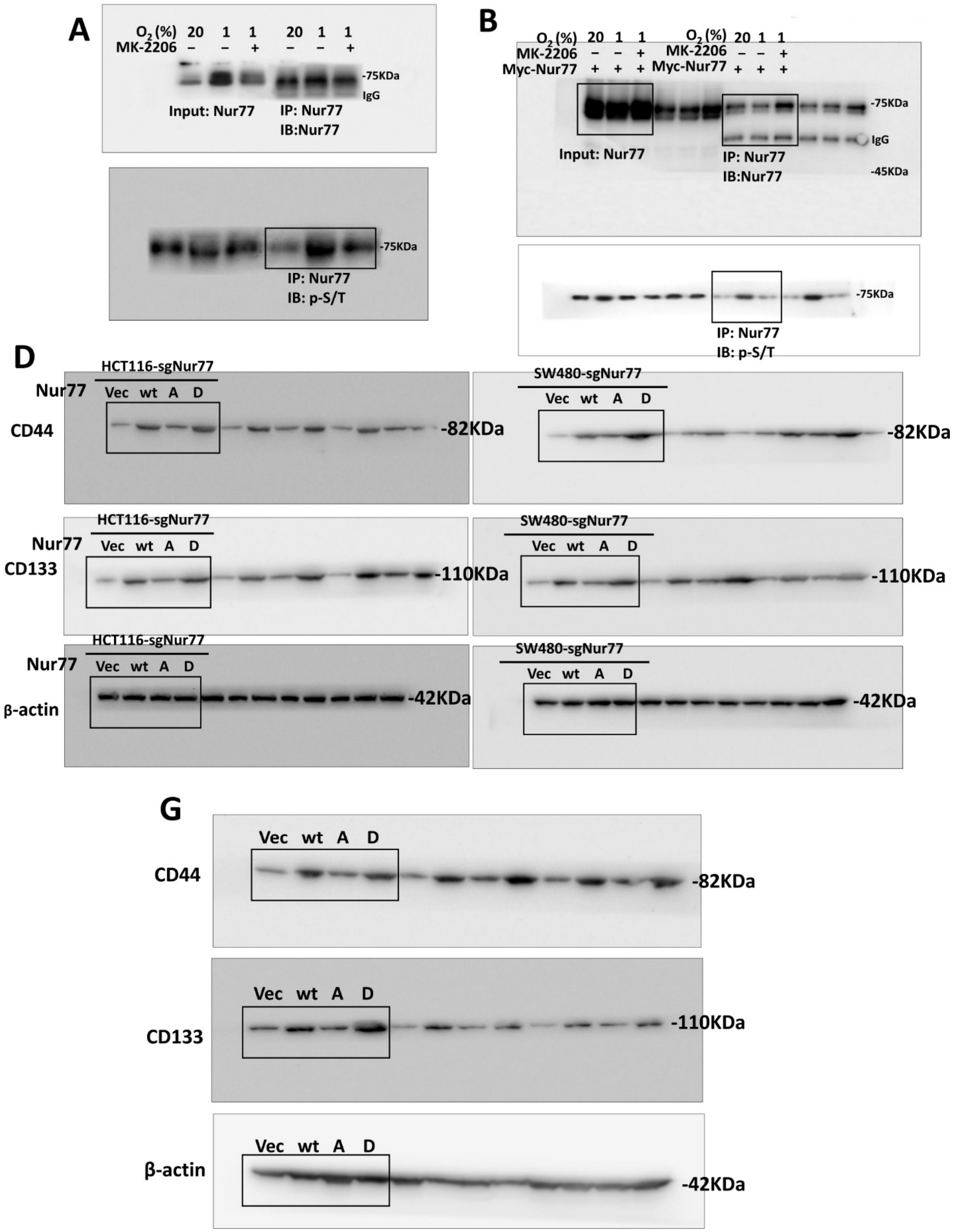

Figure S5. Uncropped Western Blot image for Figure 3.

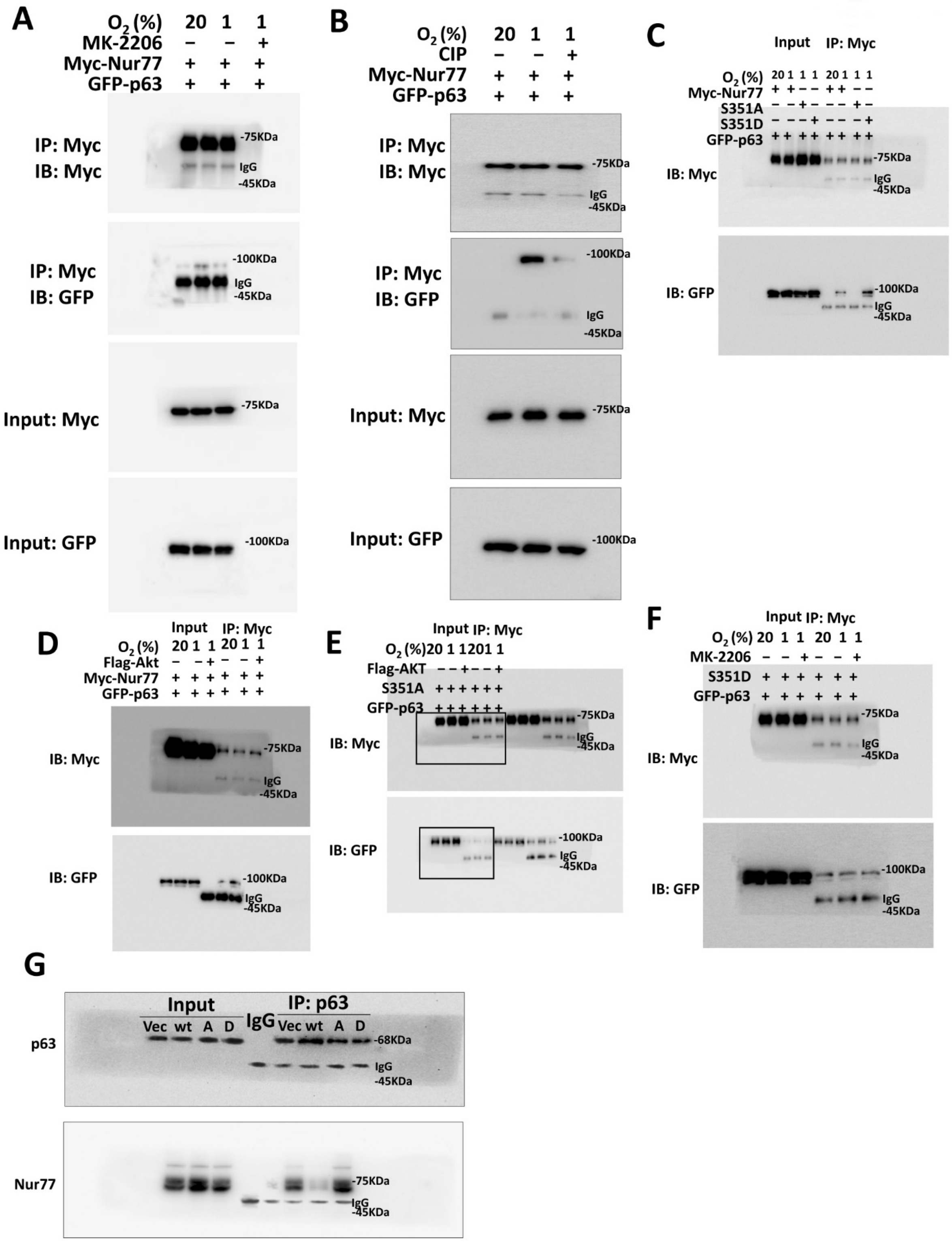

Figure S6. Uncropped Western Blot image for Figure 4.

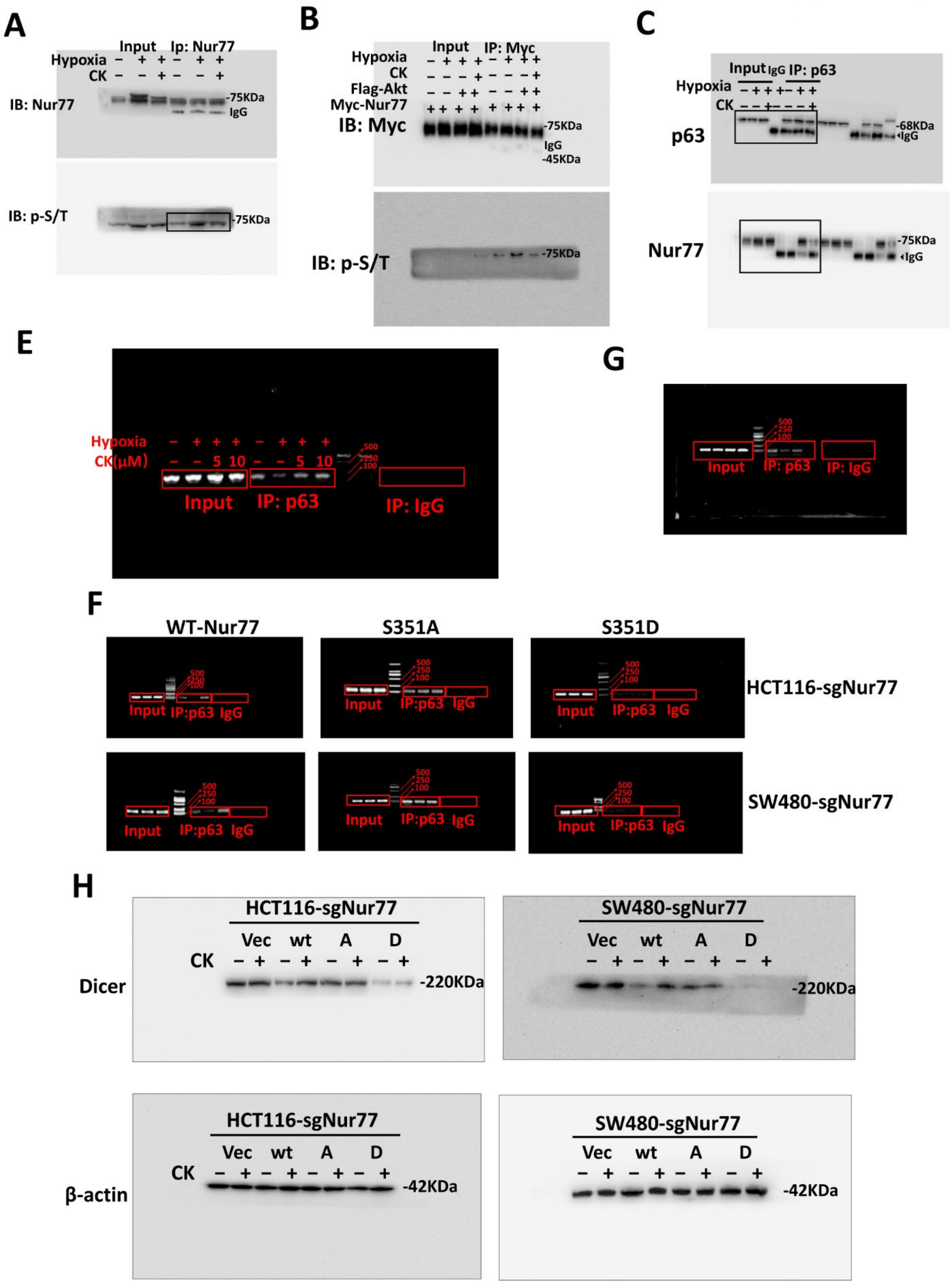
A
Input Ip: Nur77
Hypoxia
CK
IB: Nur77
-75KDa
IgG
IB: p-S/T
-75KDa
B
Input IP: Myc
Hypoxia
CK
Flag-Akt
Myc-Nur77
IB: Myc
-75KDa
IgG
-45KDa
IB: p-S/T
-75KDa
C
Input IgG IP: p63
Hypoxia
CK
p63
-68KDa
IgG
Nur77
-75KDa
IgG
E
Hypoxia
CK(μM)
5 10 5 10
500
250
100
Input
IP: p63
IP: IgG
G
500
250
100
Input
IP: p63
IP: IgG
F
WT-Nur77
S351A
S351D
Input IP:p63 IgG
HCT116-sgNur77
SW480-sgNur77
H
HCT116-sgNur77
SW480-sgNur77
Vec wt A D
CK
Dicer
-220KDa
β-actin
-42KDa

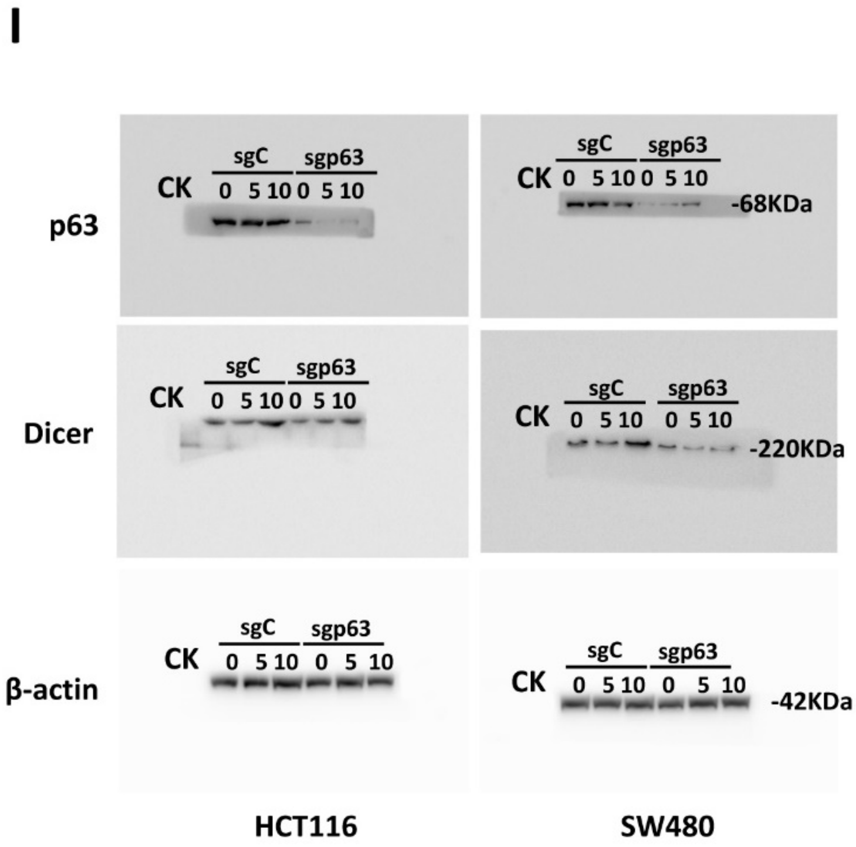

Figure S7. Uncropped Western Blot image for Figure 5.

B

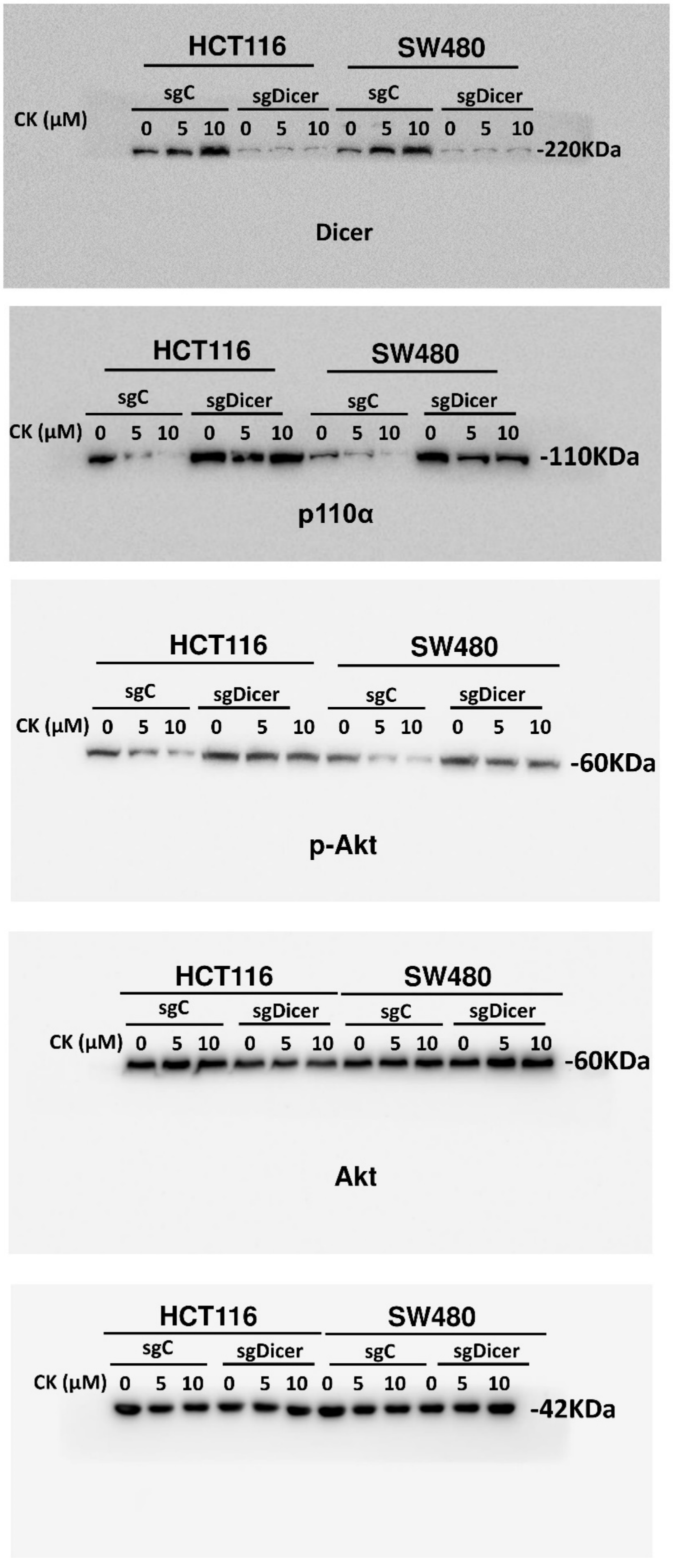
HCT116
SW480
sgC
sgDicer
CK (μM)
0 5 10 0 5 10 0 5 10 0 5 10
-220KDa
Dicer
-110KDa
p110α
-60KDa
p-Akt
Akt
-42KDa

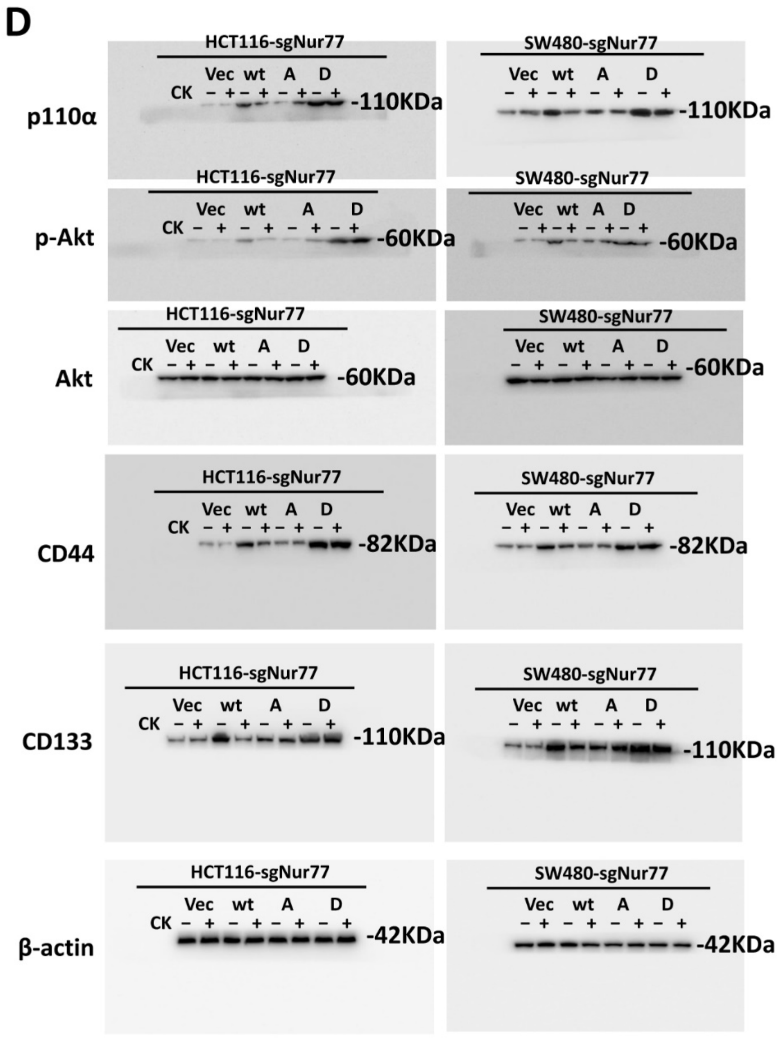

Figure S8. Uncropped Western Blot image for Figure 6.

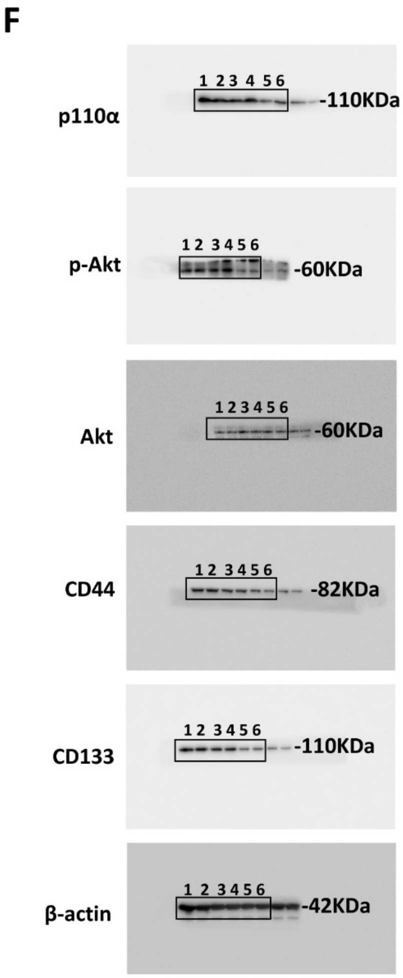

Figure S9. Uncropped Western Blot image for Figure 7.

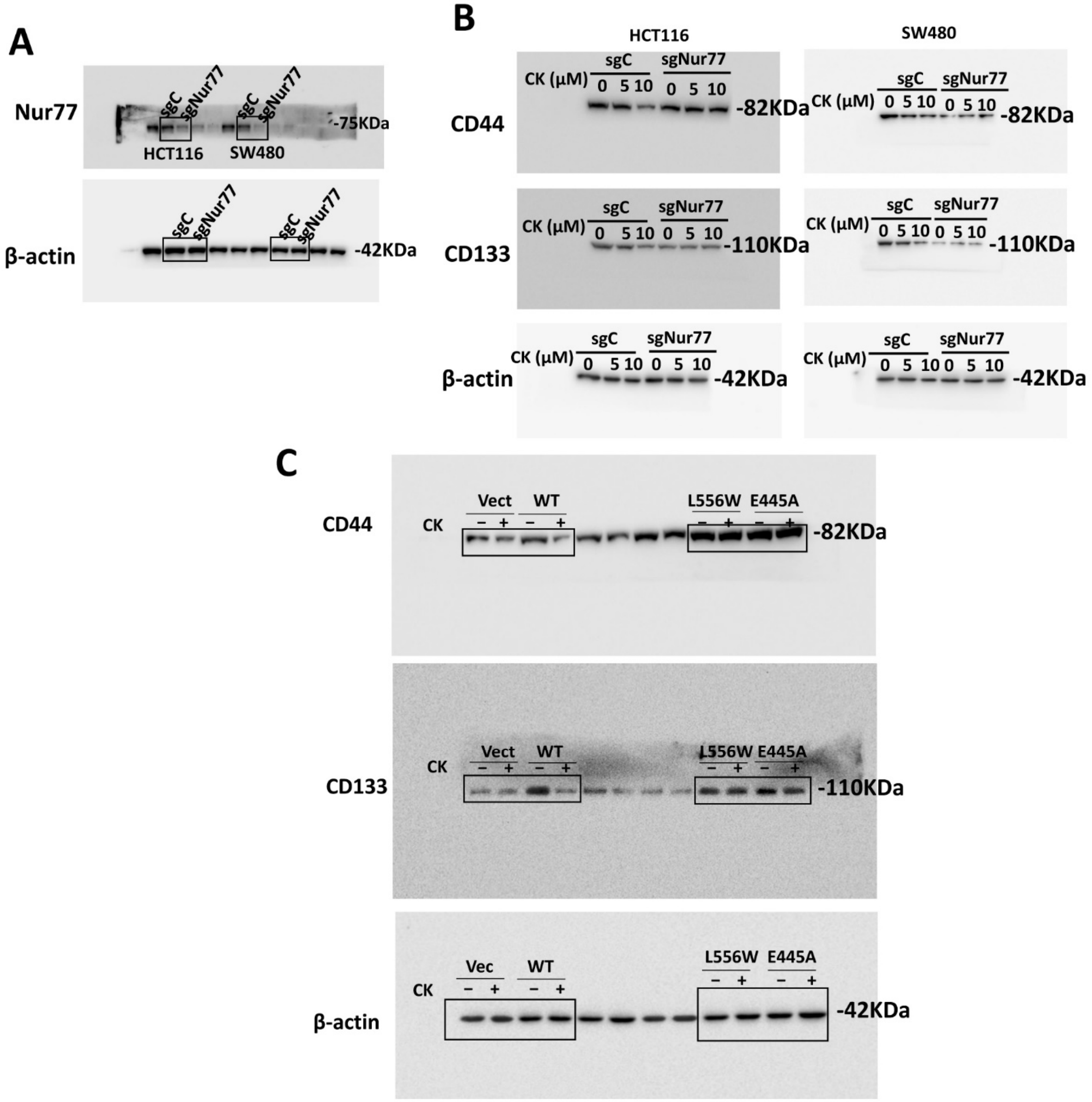

Figure S10. Uncropped Western Blot image for Figure S1.

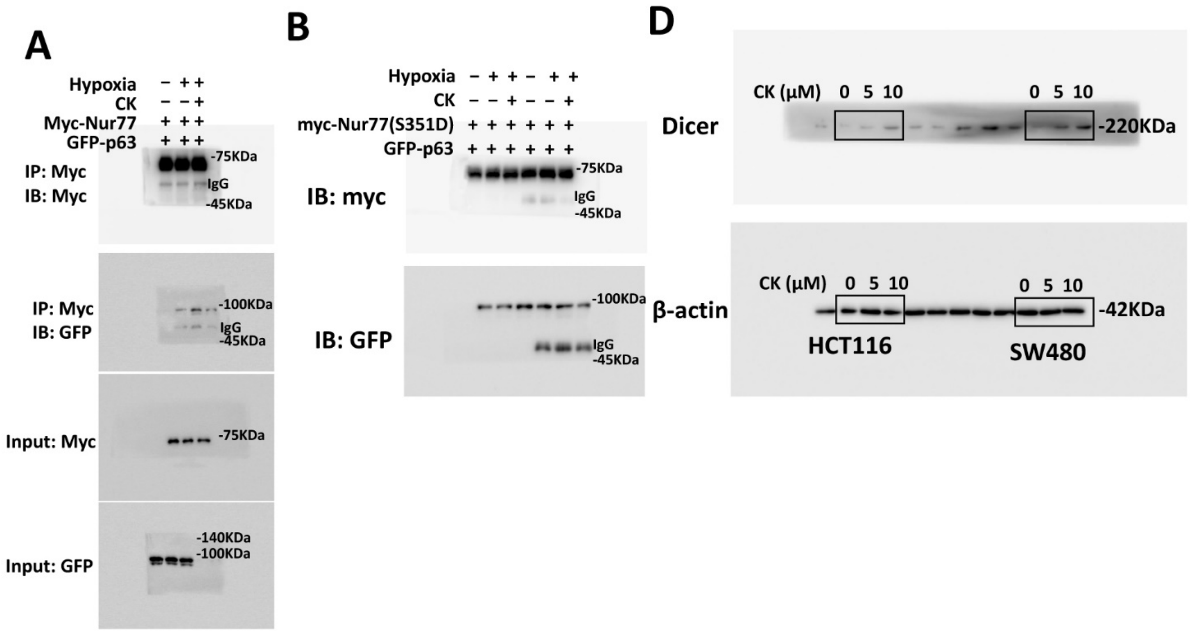

Figure S11. Uncropped Western Blot image for Figure S2.
